# Mitigation of Fumonisin Biomarkers by Green Tea Polyphenols in a High-Risk Population of Hepatocellular Carcinoma

**DOI:** 10.1038/srep17545

**Published:** 2015-12-02

**Authors:** Kathy S. Xue, Lili Tang, Qingsong Cai, Ye Shen, Jianjia Su, Jia-Sheng Wang

**Affiliations:** 1 Department of Environmental Health Science, University of Georgia, Athens, GA 30602, USA; 2 Complex Carbohydrate Research Center, University of Georgia, Athens, GA 30602, USA; 3 Department of Epidemiology and Biostatistics, University of Georgia, Athens, GA 30602, USA; 4 Guangxi Cancer Institute, Nanning, Guangxi, 530021, China

## Abstract

Green tea polyphenols (GTP) are highly effective in inhibiting a variety of tumorigenic effects induced by carcinogens. In this study we assessed GTP mitigation on biomarkers of fumonisin B_1_ (FB_1_), a class 2B carcinogen, in blood and urine samples collected from an intervention trial. A total of 124 exposed people were recruited and randomly assigned to low-dose (GTP 500 mg, n = 42), high-dose (GTP 1,000 mg, n = 41) or placebo (n = 41) for 3 months. After one-month of intervention, urinary FB_1_ was significantly decreased in high-dose group compared to that of placebo group (p = 0.045), with reduction rates of 18.95% in the low-dose group and 33.62% in the high-dose group. After three-month intervention, urinary FB_1_ showed significant decrease in both low-dose (p = 0.016) and the high-dose (p = 0.0005) groups compared to that of both placebo group and baseline levels, with reduction rates of 40.18% in the low-dose group and 52.6% in the high-dose group. GTP treatment also significantly reduced urinary excretion of sphinganine (Sa), sphingosine (So), and Sa/So ratio, but had no effect on serum Sa, So, and Sa/So ratio. Analysis with mixed-effect model revealed significant interactions between time and treatment effects of GTP on both urinary free FB_1_ levels and Sa/So ratios.

Fumonisins (FNs) are a family of naturally occurring mycotoxins mainly produced by *Fusarium verticilioides*^1^. They are ubiquitous contaminants of cereal grains worldwide, with at least 15 variants isolated, including A, B, C and P variants^2^. Fumonisin B_1_ (FB_1_), the representative mycotoxin, causes several fatal animal diseases, including leukoencephalomalacia in horses, pulmonary oedema in swine, neural tube defects in mice, nephrotoxicity in rodents, and hepatotoxicity in horses, swine and rats^3^,^4^,^5^,^6^,^7^. In addition, FB_1_ is a carcinogen and strong tumor promoter in animal and human cell models^8^,^9^,^10^,^11^,^12^.

Human populations in certain areas of the world are exposed to higher levels of FB_1_ in their daily diets, mainly through ingestion of contaminated corn and corn-based foods^2^,^13^,^14^,^15^,^16^. Aetiologically, FB_1_ exposure has been linked to a broad spectrum of human diseases, such as hepatocellular carcinoma (HCC) and oesophageal cancer (EC) in South Africa, China and the Islamic Republic of Iran^12^,^13^,^14^,^15^,^16^. The International Agency for Research on Cancer (IARC) has classified FB_1_ as a group 2B carcinogen^12^. In addition to the carcinogenic property, FB_1_ exposure played a role in the occurrence of a cluster of neural tube defects along the Texas–Mexico border^17^.

Several studies have pinpointed the possible mechanism of action of FB_1_ by means of ceramide synthase inhibition and global disruption of lipid metabolism^18^,^19^,^20^. FB_1_ disrupts sphingolipid metabolism by inhibiting ceramide synthases, due to its structural similarity to long-chain sphingoid base backbones^18^. The inhibition of ceramide synthases causes an increase in intracellular free sphinganine (Sa) and, to a lesser extent, sphingosine (So), which precedes the depletion of complex bioactive lipids^19^,^20^. FB_1_-induced biochemical alterations, particularly the elevated Sa levels, Sa to So ratio (Sa/So) or Sa 1-phosphate to So 1-phosphate (SaP/SoP) ratio in tissues, urine and blood, have been proposed as potential biomarkers in various animal species, including foals, pigs, mink, rodents, vervet monkeys, and ducks^15^,^21^,^22^. However, the correlation between these biomarkers and human exposure levels remain conflicted in different reported population studies in different areas^20^,^21^,^22^.

Toxicokinetic data showed that orally dosed FB_1_ was eliminated rapidly from circulation^15^,^21^,^22^,^23^. This rapid elimination and low bioavailability, as well as the lack of a major metabolite, indicate that FB_1_ in biological fluids may be measured directly as a possible biomarker of FB_1_ exposure^21^,^22^. Efforts have been made to monitor free FB_1_ in human urine^24^,^25^,^26^, plasma^26^, faeces^27^ and hair^28^,^29^. Currently available data demonstrated that free FB_1_, as an exposure biomarker, has been widely applied in many studies to track FB_1_ e in humans^24^,^25^,^26^,^30^,^31^,^32^,^33^. Furthermore, this biomarker has been applied to evaluate intervention strategies that could reduce exposure to FB_1_^34^,^35^.

The intervention strategies for FNs mainly focus on pre- and post-harvest control. However, the greater challenge now is to reduce the risk in individuals who have already been exposed to FNs for decades via diets. Chemoprevention was proposed as a promising strategy to help these high-risk individuals^36^. Green tea polyphenols (GTP), major components in green tea leaves, have been shown to be highly effective in inhibiting a variety of carcinogen-induced tumorigenesis in animal models at different target organ sites^36^,^37^,^38^,^39^,^40^. Previous studies from our laboratory found that GTP effectively modulated aflatoxin B_1_ (AFB_1_) metabolism as well as metabolic activation, as demonstrated by the decreased serum levels of AFB_1_-albumin adducts and urinary levels of AFM_1_ in high-risk individuals of liver cancer^41^. Further, GTP supplement effectively reduced levels of 8-hydroxydeoxyguanosine, the oxidative DNA damage biomarker, in populations exposed to high levels of AFB_1_^42^. Given the co-contamination of FB_1_ with AFB_1_ found in food samples from several study populations, as well as the synergistic toxicity found in animal models^14^,^43^,^44^, chemopreventive measures such as the supplement of GTP may also potentially modulate the FB_1_-induced toxic effects, and thus mitigate the biomarker levels of FB_1_. This hypothesis has been tested in the current study via measurement of FB_1_ biomarkers, i.e. urinary-free FB_1_, urinary and serum Sa, So, Sa/Sa ratio in samples collected from an intervention study participants with GTP in Fusui county, a high-risk area for liver cancer in China, where high levels of co-contamination of AFB_1_ and FB_1_ in food staples were recorded^13^,^14^.

## Results

### Background survey of FB_1_ exposure

Before recruitment, a total of 90 corn and corn meal samples were collected from 45 households in two study villages. FB_1_ was detectable in 95.6% (86/90) of samples with average level of 1,270.23 ± 1,490.59 μg/kg (mean ± SD), as shown in Table 1, and median level of 402.35 μg/kg. Based on the recorded food consumption in these households and detected FB_1_ levels in food, the average daily FB_1_ intake was estimated to be 750.71 ± 1,983.57 (mean ± SD) μg/kg body weight, and the median was estimated to be 138.6 (93.9–495.3) μg/kg body weight. Background levels of FB_1_ biomarkers were obtained via analyzing portions of the screening samples (n = 250). The detectable rate of urinary free FB_1_ was 91.2% (228/250), with average level of 797.66 ± 716.76 (mean ± SD) pg/mg creatinine, and median level of 560.73 pg/mg creatinine. Sphingolipids were detectable in 100% (250/250) of screening samples, and the average levels, median, lower quartile, and upper quartile of Sa, So and Sa/So ratio in the serum and urine samples were shown in Table 1.

### Baseline levels of FB_1_ biomarkers in different treatment arms

**Figure 1:**
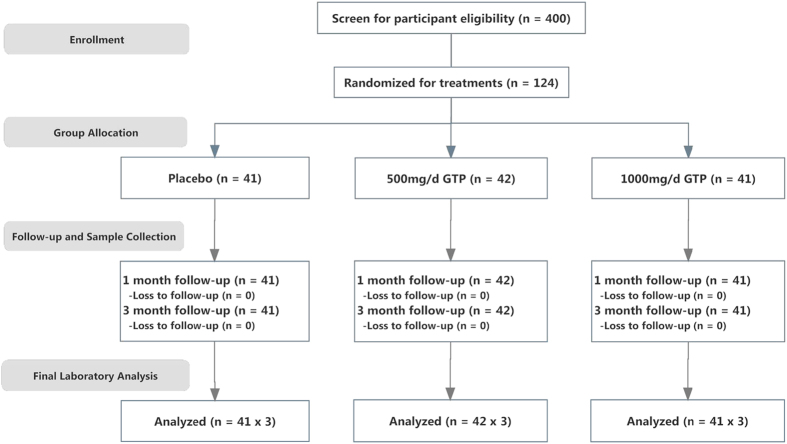
Overall study design and study outline.

As demonstrated in Fig. 1, a total of 124 subjects were recruited and randomized into three different treatment arms. Blood and urine samples were collected before the treatment for determining the baseline levels of FB_1_ biomarkers, including urinary free FB_1_, urinary and serum Sa, So and Sa/So, which were shown in Table 2. Geometric means and 95% confident intervals were also given in Table 2. Because data was not normally distributed, log-transformation was performed for statistical evaluation. Levels for each FB_1_ biomarker in three treatment arms were comparable, with no significant differences among placebo, low-dose and high-dose groups (p = 0.673, p = 0.887 and p = 0.787, respectively).

### Modulation of GTP on urinary free FB_1_ levels

**Figure 2:**
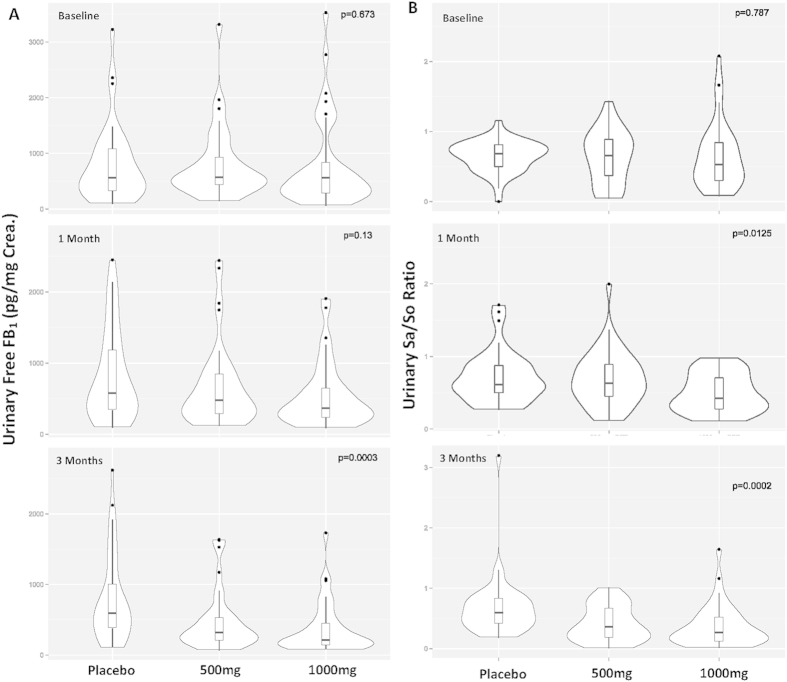
Summaries of Urinary free FB_1_ levels and Sa/So ratios **A**: Urinary free FB_1_ levels (pg/mg creatinine) in each treatment arm at the baseline, 1-month, and 3-months of GTP intervention study. **B**: Urinary Sa/So ratio in each treatment arm at the baseline, 1-month, and 3-months of GTP intervention study. The violin plot represents the overall distribution of measured data and the overlapping box plot shows the median, 25^th^ and 75^th^ percentiles of the data.

After one month of GTP intervention, the median levels of urinary free FB_1_ were 575.25, 477.79 and 364.94 pg /mg creatinine in placebo, low-dose and high-dose GTP groups, respectively. The reduction rate was 18.95% in the low-dose group and 33.62% in the high-dose group as compared to the placebo group (Fig. 2A). The urinary free FB_1_ level in the high-dose group was significantly lower than that in the placebo group (p = 0.045).

After three months of intervention, urinary free FB_1_ levels continuously decreased in GTP intervention groups, with median levels of 319.45 and 215.83 pg/mg creatinine in the low- and high-dose groups, respectively, as compared to the placebo group (591.24 pg/mg creatinine). The reduction rate was 40.18% (p = 0.016) in the low-dose group and 52.6% (p = 0.0005) in the high-dose group, both of which had significant statistical differences compared to placebo group (Fig. 2A).

### Effects of GTP on urinary sphingolipids levels

After one month of GTP intervention, the median levels of urinary Sa, So and Sa/So ratio were 3.04 nmol/L, 5.07 nmol/L and 0.63 in the low-dose group, which had no statistical significance compared to 3.85 nmol/L, 5.65 nmol/L and 0.62 in the placebo group (Fig. 2B). However, a 30.65% reduction (0.43) in Sa/So ratio was found in the high-dose group, which was statistically significant (p = 0.012) (Fig. 2B).

After three months of GTP intervention, the median levels of urinary Sa, So and Sa/So ratio were 2.32 nmol/L, 6.38 nmol/L and 0.36 in the low-dose group. Sa level and Sa/So ratio were significantly lower than those in the placebo group (3.61 nmol/L and 0.58), with reduction rates of 35.73% (p = 0.049) and 37.93% (p = 0.008), respectively, when compared to placebo group. No significant difference was found for So levels (5.56 vs 6.38 nmol/L; p > 0.05). The median levels of urinary Sa, So and Sa/So ratio were 1.56 nmol/L, 3.88 nmol/L and 0.26 in the high-dose group, with reduction rates of 56.79% (p = 0.0003), 30.22% (p = 0.23) and 55.17% (p = 0.0002), respectively, when compared to placebo group (Fig. 2B).

### Effect of GTP on serum sphingolipid levels

**Figure 3:**
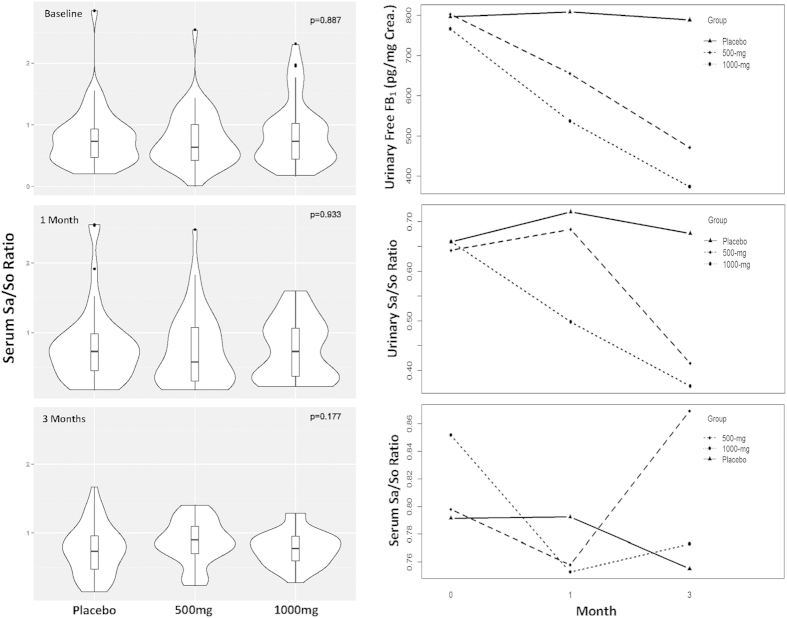
Summaries of serum Sa/So ratios and treatment/time interaction **A:** Serum Sa/So ratio in each treatment arm at the baseline, 1-month, and 3-months of GTP intervention study. The violin plot represents the overall distribution of measured data and the overlapping box plot shows the median, 25^th^ and 75^th^ percentiles of the data. **B:** Plots of interaction between group and time generated by the mixed-effect model for three biomarkers, urinary free FB_1_ levels, urinary Sa/So ratio, and serum Sa/So ratio, in three treatment arms of GTP intervention study.

After one month of GTP intervention, the median levels of serum Sa, So and Sa/So ratio were 10.06 nmol/L, 16.42 nmol/L and 0.65 in the low-dose group, which had no statistical significances when compared to 14.11 nmol/L, 20.31 nmol/L and 0.73 in the placebo group. The median levels of serum Sa, So and Sa/So ratio were 13.1 nmol/L, 16.86 nmol/L and 0.73 in the high-dose group, which also had no significant differences with the placebo group (Fig. 3A).

After three months of GTP intervention, the median levels of serum Sa, So and Sa/So ratio were 11.37 nmol/L, 13.79 nmol/L and 0.90 in the low-dose group, which had no statistical significances when compared to 11.32 nmol/L, 13.34 nmol/L and 0.73 in the placebo group. The median levels of serum Sa, So and Sa/So ratio were 7.5 nmol/L, 9.5/nmol/L and 0.76 in the high-dose group. The reduction rates were 33.75% (p = 0.038) for Sa levels and 28.79% (p = 0.045) for So levels, which were statistical significant when compared to the placebo group, but the Sa/So ratio (0.76 vs 0.73) was not significantly different (Fig. 3A).

### Interactions between time and treatment

A log-transformed mixed–effect model was used to analyse interactions between time and treatment effects, and the outcomes were shown in Table 3 and Fig. 3B. The interaction between time and treatment effects on urinary free FB_1_ was linear and highly significant (p = 0.0027 in the low-dose group and p = 0.0006 in the high-dose group). Interaction between time and treatment effects on urinary Sa/So ratio was also significant, with p = 0.0387 in the low-dose group and p = 0.02 in the high-dose group, respectively. However, no interaction between time and treatment effects was found on serum Sa/So ratio, as demonstrated in both Table 3 and Fig. 3B.

## Discussion

The key finding from this study was that urinary biomarkers, free FB_1_ and Sa/So ratio, decrease significantly in GTP treatment groups, and the effect was greater over time, which demonstrated the mitigative effect of GTP on FB_1_ exposure in this high-risk human population of HCC. The urinary free FB_1_ reflects the amount absorbed into circulating system, which was found to be the most sensitive biomarker among all FB_1_ biomarkers examined. Gong *et al.*^30^ showed the correlation of urinary FB_1_ with maize intake in a Mexican population. Similarly, van der Westhuizen *et al.*^31^ found urinary FB_1_ level reflected changes in FNs exposure in Centane, South Africa, when an intervention measure was applied to the population to reduce FN intake. Torres *et al.*^33^ have further confirmed that urinary free FB_1_ mirrored estimated FB_1_ intake, by assessing urinary and maize samples collected from high and low exposure communities in Guatemala. In addition, Shirima *et al.*^32^ have used urinary free FB_1_ level as biomarker to assess the exposure among Tanzanian children.

FB_1_ is an ubiquitous food contaminant, which acts as a competitive inhibitor to ceramide synthase, thus disrupting synthesis of complex sphingolipids, resulting in accumulation in the levels of free sphingolipids such as sphingosine and sphinganine^15^,^18^,^19^,^20^,^21^. Our laboratory had previously focused on validating various FB_1_ biomarkers, including FB_1_-induced changes in sphingolipid metabolism as well as frees FB_1_^22^,^26^. Urinary Sa/So ratio was found to be more sensitive than serum Sa/So ratio in F344 rats in response to both acute and sub-chronic exposure to FB_1_. Free FB_1_ excretions in urine and faeces were strongly correlated with urinary sphingolipid metabolites in the repeat-dose study^22^. Findings from the cross-sectional study supported the notion that humans were exposed to higher levels of dietary FNs in the high-risk areas of oesophageal cancer and HCC in China, as compared to the low-risk area of both cancers^13^. Moreover, higher estimated FB_1_ daily intakes could be mirrored by elevations of urinary free FB_1_ and urinary Sa/So ratio among residents of those high-risk areas^26^. Longitudinal biomonitoring study also demonstrated temporal variations in urinary and serum Sa/So ratios, which reflected the seasonal changes of FB_1_ intake and food contamination in the high-risk areas of oesophageal cancer and HCC^26^.

As demonstrated by various animal studies^20^,^21^, the alterations in the sphingolipid levels as well as ratios were direct consequences of lipid profile changes induced by FB_1_. The decrease in the levels of urinary free FB_1_ and Sa/So ratio pinpoint to a decreased level of FB_1_ absorption, as well as reduced toxicity, when treated with GTP. It should be noted that, while the urinary Sa/So ratio displayed statistically significant decrease over time in treated groups, the serum Sa/So ratios did not. Levels of Sa, So and Sa/So ratio in blood had previously been proposed as potential biomarkers due to their association with treatment in animal models as well as the mechanistic implication; however, the reliability of these biomarkers in human populations remains questionable, mainly due to the influence of other factors and inconsistency in human studies^21^. For example, Solfrizzo *et al.*^45^ found that Sa/So ratio in South Brazil population was significantly higher than that of North Argentina, despite of similar maize and FNs intake in the two areas. Similar phenomenon was found by van der Westhuizen *et al.*^46^, in assessing Sa and So levels in different areas of former Transkei, South Africa. They found no association between levels of Sa, So and/or Sa/So ratio and dietary levels of FB_1_ in both urine and plasma samples in area of low and high oesophageal cancer incidences in Transkei region of South Africa^47^. Others, as well as our previous studies, also found neither between FB_1_ exposure and serum Sa/So ratio^26^, nor association between serums Sa/So ratio and oesophageal cancer risk^48^. One hypothesis for this lack of correlation may be due to role of blood stream in buffering sphingolipids. Because new sphingolipids were continually synthesized *de novo* and released into bloodstream, this would in turn result in high variation in the sphingolipid levels. Moreover, the two sphingolipids were affected differently by the salvage pathway of sphingolipid metabolism, which may also cause discrepancies in serum ratio.

There have been great concerns in recent years on co-contamination of AFB_1_ and FB_1_ in cereal grains and potential health risks of dietary co-exposure to these two mycotoxins^14^. Co-contamination of AFB_1_ and FB_1_ in dietary components, especially in corn and corn products, has been reported worldwide^14^,^49^,^50^,^51^,^52^. US corn and corn products were found with detectable levels of AFB_1_ in 0.5 to 200 ppb and FB_1_ in 0.5 to 150 ppm^53^. Synergistic interactions on toxicity and co-carcinogenicity were found in various animal models co-exposed to AFB_1_ and FB_1_, including in trout, rats, chicks, and mosquito fish^43^,^44^,^54^,^55^,^56^. Co-exposure to these two mycotoxins has also been suggested to be linked with the aetiology of acute mycotoxicosis, reproductive and developmental disorders, as well as primary liver cancer and oesophageal cancer^14^. It has been well known that AFB_1_ is potent genotoxic agent, and genetic alterations played important roles in its mode of actions, whereas FB_1_ is not genotoxic, and its tumorigenic effect is believed to be mainly via epigenetic mechanisms, including disruptions of apoptosis, cell cycle, and sphingolipids signalling pathways.

While molecular mechanisms related to the synergistic adverse health outcomes induced by co-exposure are under investigation, several intervention strategies targeting co-exposure to AFB_1_ and FB_1_ have been devised and studied. Given the source of mycotoxin production, sorting and cleaning up has shown to effectively reducing FB_1_ and AFB_1_ exposure^31^. Alternatively, conjugation of AFB_1_ and FB_1_ with enterosorbents may prevent absorption, thereby reducing internal dose and toxic effects. Calcium montmorillonite clay in diet was shown to effectively bind to AFB_1_, and to some extent FB_1_, reducing the absorption of both, as well as urinary excretion of the biomarkers^34^,^55^. Another intervention involves chemically modifying these two mycotoxins in food source via alkaline treatment (nixtamalization), which greatly reduced the amount of AFB_1_ and FB_1_ in food and significantly reduced toxicity observed^4^,^56^. Effectiveness of this strategy in reducing human co-exposure and toxicity is in active investigation.

By comparison, we previously found chemopreventive effect of GTP in reducing AFB_1_ biomarkers^41^ in the same population, and this study demonstrated a mitigation of FB_1_ exposure via reducing FB_1_ biomarkers, rather than by a physical means of intervention. While the direct binding of GTP to AFB_1_ and FB_1_ seems unlikely, GTP modulated the expression of key enzymes involved in Phase 2 detoxifying enzymes or ceramide synthesis pathway, both of which played significant roles in AFB_1_ and FB_1_ toxicity and carcinogenicity^36^,^37^,^38^,^39^,^40^,^41^,^42^. GTP modulated apoptosis and cell cycle regulating genes in animals and human cells^27^,^28^,^29^,^30^. As potent antioxidants, GTP may also play a role in maintaining the integrity of cell membrane and preventing lipid oxidation, which may mitigate the toxic effects of FB_1_.

The toxicokenetics of FB_1_ were characterized by low absorption, rapid distribution and elimination, and a lack of phase 1 metabolism in animal models *in vitro* or *in vivo*^21^. Because of the absence of measurable metabolites, free FB_1_ itself was proposed as a biomarker^20^,^21^,^22^. Kinetics study showed that urinary excreted FB_1_ consists of average 0.5–1% of dietary intake, and would be eliminated from the system rapidly^22^. The enteric track plays a major role in limiting FB_1_ absorption, as it is the primary entry into bloodstream for both AFB_1_ and FB_1_. In cell model, enteric cells are shown to limit FB_1_ absorption, in particular, the P-glycoprotein and multidrug resistance associated proteins, which, when inhibited, significantly increases the passage of FB_1_ through the cells^57^. Co-exposure to AFB_1_ and FB_1_ may potentially affect the enteric cellular structure, resulting in increased absorption of both AFB_1_ and FB_1_^58^. Treatment with GTP, on the other hand, reduces the oxidative effect, allowing preservation of the integrity of enteric barrier, therefore mitigating the exposure to both toxins. Given the potential role of the enteric system involved in absorption and metabolism of AFB_1_ and FB_1_, the enteric system can be a potential target for chemopreventive measures, both in terms of structural integrity and the gut micro-environment. GTP can promote the growth and maintenance of gut microbiota^59^, potentially modulate the absorption and metabolism of AFB_1_ and FB_1_.

In summary, GTP can effectively mitigate urinary FB_1_ biomarkers in a high risk population via several mechanisms as discussed above, and supplement of GTP can potentially be a useful chemopreventive strategy for reducing co-exposure to AFB_1_ and FB_1_.

## Materials and Methods

### Chemicals

GTP used for the study was obtained from the US-China joint venture Shili Natural Product Company, Inc. (Guilin, Guangxi, China) and the purity of GTP is higher than 98.5% according to the analysis by Guangxi Standard Bureau. Boric acid, D-erythro-sphingosine, D-erythro-sphinganine, FB_1_ from *F. verticilioides* (approximately 98% by HPLC), formic acid, hydrochloric acid, 2-mercaptoethanol, O-phthaldialdehyde (OPA), 10 x phosphate-buffered saline (PBS), and sodium phosphate monobasic were purchased from Sigma-Aldrich (St. Louis, MO, USA). D-erythro-C_20_-sphingosine was obtained from Avanti Polar Lipids, Inc. (Alabaster, AL, USA). Triethylammonium formate HPLC buffer (pH 3.0) was purchased from Regis Technologies, Inc. (Morton Grove, IL, USA). Other HPLC-grade solvents including acetonitrile, ethyl acetate, methanol, 2-propanol, and water were from Honeywell Burdick & Jackson (Muskegon, MI, USA). OPA reagents were prepared by dissolving 10 mg of OPA and 30 μl of 2-mercaptoethanol in 250 μl of methanol and mixing with 4.75 ml of 3% boric acid buffer (pH 10.5).

### Study sites, subjects and protocols

The study site included two villages (Sanhe and Zhuqing), located 45 km southwest of Fusui county, Guangxi Zhuang Autonomous Region, China. The site is a rural farming community with 7500 residents, and it belongs to the Qujiu Township, one of the three townships with the highest incidence and mortality of liver cancer (approximately 100/100 000) in Fusui county. The overall study design was previously described in detail^36^ and outlined in Fig. 1. The study was approved by the Institutional Review Boards of University of Georgia and Guangxi Cancer Institute for human subject protection, and was carried out in accordance with the corresponding guidelines.

Participant inclusion criteria included urinary and serum FB_1_ biomarker-positive adults aged 20–55, with normal liver and kidney function tests, alpha-fetoprotein negative, no personal history of cancer, no use of prescribed medication, and no pregnancy and lactation for female participants. Informed consent was obtained from all study participants. Four-hundred adults were screened via survey with a short questionnaire, physical exam, and clinical and biomarker analysis of their blood and urine samples. Those who didn’t meet criteria were excluded. A total of 124 healthy eligible adults aged 20–49 years were recruited, about one third (41/124) were females. The participants were randomized into three groups, each treated daily with placebo, 500 mg GTP (low-dose), or 1000 mg GTP (high-dose) capsules for 3 months. The low and high doses were chosen to be equivalent of consumption of two and four cups of green tea consumption, respectively, which are reflective of typical daily intake level in common people. The GTP treatments consist of 250 mg GTP capsules, each containing 126 mg epigallocatechin gallate (EGCG), 53 mg epicatechin gallate (ECG), 25 mg epicatechin (EC), 19 mg epigallocatechin (EGC), 14 mg gallocatechin gallate (GCG) and 11 mg catechin (C), according to HPLC-ECD and HPLC-UV analysis by Guangxi Standard Bureau and our laboratory. Participants were arranged to take capsules twice a day, both after meal.

In addition to regular epidemiological questionnaires, blood samples (7 ml) were drawn after overnight fast, before the treatment (as the baseline), and at 1 month and 3 months of the GTP supplement. Serum was separated via centrifugation at 2500 rpm for 15 min and immediately stored at −20 °C. Twenty-four hour urine samples were collected in the morning, noon and evening of the same day, and stored in amber bottles containing ascorbic acid (20 mg/mL) and EDTA (0.1 M). Aliquots of urine samples (50 ml) were treated with 500 mg ascorbic acid and 12.5 mg EDTA for GTP components and biomarker analysis. All samples were shipped frozen to Guangxi Cancer Institute at Naning City, and further shipped in frozen via air to US laboratory. Laboratory personnel who performed analysis were blinded to sample sources.

Follow-up visits to the participants’ houses were made every other day to record adverse-effects, as well as to assess adherence to treatment. Clinical tests were conducted during each sample collection, including blood counts, blood chemistry, urinary protein, glucose, and blood, which showed no severe adverse-effects^36^. Excellent person- time compliance (99.5%) was achieved, and no consumption of tea or tea products or broccoli outside of treatments assigned was reported for any participant in this trial. Sample collection, storage and shipment complied with guidelines of both Chinese and US governments.

### Food samples collection and FB_1_ analysis

Corn or corn meal samples (500 g/item) were collected from individual households of the study villages before recruitment. The FB_1_ level in food samples was measured using the enzyme-linked immunosorbent assay (ELISA) kit from Neogen Corp. (Lansing, MI, USA) according to the manufacturer’s procedures. The limit of detection is 0.05 mg/kg.

### Sample size determination

Data from previous studies^26^ and screening study was used for calculation of sample size. Equal sample size for each treatment group (placebo, low-dose and high-dose GTP) was assumed. The primary objective is to test if FB_1_ biomarker concentrations will be affected by GTP intervention. According to the mean and standard deviation (SD) values for the primary outcome of urinary FB_1_ (ng/mg creatinine) found in previous studies, the sample size was calculated based on detecting a 20% change in urinary FB_1_, as compared to the placebo group, with a power of 0.8 at α  =  0.05. An assumed correlation coefficient of 0.80 between baseline and follow-up measurements within a treatment group was used. To meet the statistical power, the sample size for each group was estimated to be 35, which requires 105 participants at an attrition rate of 10–15%.

### Urinary free FB_1_ extraction and analysis with HPLC and LC/MS confirmation

Frozen urine samples were extracted and purified according to the previously developed protocol with FumoniTest immunoafinity column from VICAM (Watertown, MA, USA)^12^,^16^. HPLC-fluorescence analysis was carried out on Agilent 1100 liquid chromatography system (Agilent Technologies, Wilmington, DE, USA) with the emission wavelength of 440 nm and excitation wavelength of 330 nm. An on-line automatic injector program performed the derivitization of FB_1_. Chromatographic separations were performed on a Luna C18 column (5μm particle size, 250 × 4.6 mm) from Phenomenex (Torrance, CA, USA). The mobile phase consisted of a linear gradient starting from 0.1 M sodium phosphate monobasic (pH 3.4)-methanol (35/65, v/v) to 0.1 M sodium phosphate monobasic (pH 3.4)-methanol (20/80, v/v) over 13 min, with flow rate of 1.0 ml/min and injection volume of 100 μl. The limit of quantitation for this method was 20 pg per injection. The mean recovery rate was 83.4% ± 0.8%. The relative standard deviation (RSD) was 4.42%. FB_1_ concentration was verified using LC/MS methods described in previous studies^26^. Urinary creatinine levels were analysed using the 96-well Creatinine Assay Kit from Cayman Chemical (Ann Arbor, MI, USA).

### Extraction and analysis of serum and urine sphingolipids

Extraction procedure and analysis were slightly modified from protocols of previous reports^20^,^26^. The fluorescent derivatives of So, Sa and C_20_So standard were resolved using Agilent 1100 liquid chromatography system (Agilent Technologies, Wilmington, DE, USA) with the excitation and emission wavelengths of 340 and 455 nm, respectively. An on-line antomatic injector program performed the derivitization of sphingoid bases. Separations were performed using a Zorbax Eclipse XDB-C18 column (5μm particle size, 250 × 46 mm, Agilent Technologies). The mobile phase consist of a linear gradient starting from 5 mM triethylammonium formate (pH 4.3)-methanol-acetonitrile (15/45/40, v/v/v) to methanol-acetonitrile (60/40, v/v) over 20 minutes at flow rate of 1.0 ml/min. The limit of quantitation for this method was 0.03 pmol per injection.

### Statistical analysis

The analyses comprised 3 components: (i) a description of urinary FB_1_, serum and urinary sphingolipids at the individual level, (ii) a comparison of levels of FB_1_ biomarkers by treatment arms at baseline, before the administration of the GTP and (iii) an evaluation the overall effects of GTP on FB_1_ biomarkers. The concentrations of FB_1_, serum and urinary sphingolipids (sa, so and sa/so ratio), were expressed as mean ± SD and median (interquartile range), unless otherwise stated. The levels of FB_1_ biomarker in urine were adjusted by the concentration of creatinine, and were summarized using descriptive statistics. For the baseline comparison between the treatment and placebo arms, a Wilcoxon rank-sum test for each biomarker was conducted to test if randomization achieved comparable groups. To evaluate the treatment effects, separate mixed-effect models for urinary FB_1_, Sa, So, and Sa/So ratio were constructed. The model included the intercept, indicators for treatment group, time, and a treatment by time interaction term as fixed effect terms. Then individual-level intercept and time variables were included as random effects. The model has the mathematical form as shown in equation (1):


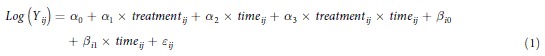


where Y_ij_ Y_ij_ is the response variable value for sample *i* measured at time *j*; *i* = 1, 2, …, N, with N being the total sample size;

*j* = month 0, 1 and 3;

*α*_*0*_, *α*_*1*_, α_2_, α_3_ α_0_, α_1_, α_2_, α_3_ are the fixed effect coefficients;

β_ί0_ *β*_*i*0_′*s* and β_ί1_ *β*_*i*1_′*s* are the random effect coefficients;

*ε*_*ij*_ is the error term.

Response variables that were not normally distributed were transformed by logarithmic to improve normality. The final mixed model was fitted using PROC MIXED in SAS software. Parameters of the mixed model were estimated using Maximum Likelihood Estimation (MLE) method. The Akaike Information Criteria (AIC) and the Bayesian Information Criteria (BIC), where smaller values for both are considered more preferable, were used as measures of the relative qualities of particular models. Both AIC and BIC dealt with the trade-off between the goodness of fit of the model and the complexity of the model, and thus provided valid means for model selection^60^. Statistical hypothesis tests were two-tailed and assumed an alpha error of 0.05. All analyses were conducted in SAS 9.3 (SAS Institute, Cary, NC, USA).

## Additional Information

**How to cite this article**: Xue, K. *et al.* Mitigation of Fumonisin Biomarkers by Green Tea Polyphenols in a High-Risk Population of Hepatocellular Carcinoma. *Sci. Rep.* **5**, 17545; doi: 10.1038/srep17545 (2015).

## Figures and Tables

**Table 1:** Background information on FB_1_ exposure in screening study subjects.

	Mean ±SE	Median (Lower Quartile, Upper Quartile)
FB_1_ Levels in household corn (mg /kg)	1.27 ± 14.91	0.4 (0.1–1.2)
Estimated daily FB_1_ intake (μg /day bw)	750.71 ± 1983.57	138.6 (93.9–495.3)
Urinary Free FB_1_ (pg/mg Crea.)	797.66 ± 716.76	560.73 (313.45, 1051.67)
Serum Sphingolipids (nmol/L)		
Sa	16.07 ± 14.30	10.34 (5.57, 23.01)
So	21.05 ± 14.07	17.95 (9.52, 31.53)
Sa/So	0.81 ± 0.48	0.71 (0.45, 1.02)
Urinary Sphingolipids (nmol/L)		
Sa	10.82 ± 18.76	3.47 (1.69, 12.19)
So	15.18 ± 20.48	5.68 (3.74, 21.19)
Sa/So	0.65 ± 0.36	0.64 (0.42, 0.84)

**Table 2:** Baseline Levels of FB_1_ biomarkers in treatment groups; GM: Geometric means.

	Placebo (GM; 95% CI)	GTP 500 mg (GM; 95% CI)	GTP 1000 mg (GM; 95% CI)	p
Number	41	42	41	
Free Urinary FB_1_(pg/mg Crea.)	575.44(436.52, 776.25)	630.96(489.78, 812.83)	512.86(380.19, 707.95)	0.673
Serum Sa(nmol/L)a	11.02(8.50, 14.30)	11.36(8.76, 14.88)	11.36(8.50, 15.18)	
Serum So(nmol/L)	16.12(12.55, 20.70)	16.28(12.81, 20.70)	15.8(12.06, 20.91)	
Serum Sa/So	0.68(0.58, 0.81)	0.69(0.59, 0.83)	0.72(0.59, 0.87)	0.887
Urinary Sa(nmol/L)	4.81(3.25, 7.10)	4.26(2.66, 6.75)	3.86(2.46, 6.11)	
Urinary So(nmol/L)	7.69(5.31, 11.13)	8.94(6.30, 12.81)	7.61(5.42, 10.70)	
Urinary Sa/So	0.63(0.56, 0.69)	0.47(0.35, 0.65)	0.5(0.39, 0.66)	0.787

**Table 3:** Significances on interactions (Pr > |t|) between time and treatment with the mixed-effect model.

Effect	Groups	urinary FB_1_	Urinary Sa/So	Serum Sa/So
Intercept		<0.0001	<0.0001	<0.0001
Groups	placebo	–	–	–
Groups	500mg	0.8587	0.9010	0.8137
Groups	1000mg	0.7939	0.5078	0.8073
Time		0.8306	0.9494	0.6992
Time * Groups	placebo	–	–	–
Time * Groups	500mg	0.0027	0.0387	0.3785
Time * Groups	1000mg	0.0006	0.0204	0.8576
